# Gut integrity in intensive care: alterations in host permeability and the microbiome as potential therapeutic targets

**DOI:** 10.1186/s40560-025-00786-y

**Published:** 2025-03-18

**Authors:** Takehiko Oami, Takashi Shimazui, Tetsuya Yumoto, Shunsuke Otani, Yosuke Hayashi, Craig M. Coopersmith

**Affiliations:** 1 Department of Surgery and Emory Critical Care Center, Emory University School of Medicine, 101 Woodruff Circle, Suite WMB 5105, Atlanta, GA 30322 USA; 2 Department of Emergency and Critical Care Medicine, Chiba University Graduate School of Medicine, Chiba, Japan; 3 Department of Emergency, Critical Care and Disaster Medicine, Faculty of Medicine, Dentistry and Pharmaceutical Sciences, Okayama University, Okayama, Japan

**Keywords:** Intestine, Barrier, Tight junction, Claudin, Occludin, Microbiota, Pathobiome, Dysbiosis, Critical care, Sepsis

## Abstract

**Background:**

The gut has long been hypothesized to be the “motor” of critical illness, propagating inflammation and playing a key role in multiple organ dysfunction. However, the exact mechanisms through which impaired gut integrity potentially contribute to worsened clinical outcome remain to be elucidated. Critical elements of gut dysregulation including intestinal hyperpermeability and a perturbed microbiome are now recognized as potential therapeutic targets in critical care.

**Main body:**

The gut is a finely tuned ecosystem comprising ~ 40 trillion microorganisms, a single cell layer intestinal epithelia that separates the host from the microbiome and its products, and the mucosal immune system that actively communicates in a bidirectional manner. Under basal conditions, these elements cooperate to maintain a finely balanced homeostasis benefitting both the host and its internal microbial community. Tight junctions between adjacent epithelial cells selectively transport essential molecules while preventing translocation of pathogens. However, critical illness disrupts gut barrier function leading to increased gut permeability, epithelial apoptosis, and immune activation. This disruption is further exacerbated by a shift in the microbiome toward a “pathobiome” dominated by pathogenic microbes with increased expression of virulence factors, which intensifies systemic inflammation and accelerates organ dysfunction. Research has highlighted several potential therapeutic targets to restore gut integrity in the host, including the regulation of epithelial cell function, modulation of tight junction proteins, and inhibition of epithelial apoptosis. Additionally, microbiome-targeted therapies, such as prebiotics, probiotics, fecal microbiota transplantation, and selective decontamination of the digestive tract have also been extensively investigated to promote restoration of gut homeostasis in critically ill patients. Future research is needed to validate the potential efficacy of these interventions in clinical settings and to determine if the gut can be targeted in an individualized fashion.

**Conclusion:**

Increased gut permeability and a disrupted microbiome are common in critical illness, potentially driving dysregulated systemic inflammation and organ dysfunction. Therapeutic strategies to modulate gut permeability and restore the composition of microbiome hold promise as novel treatments for critically ill patients.

## Background

**Fig. 1:**
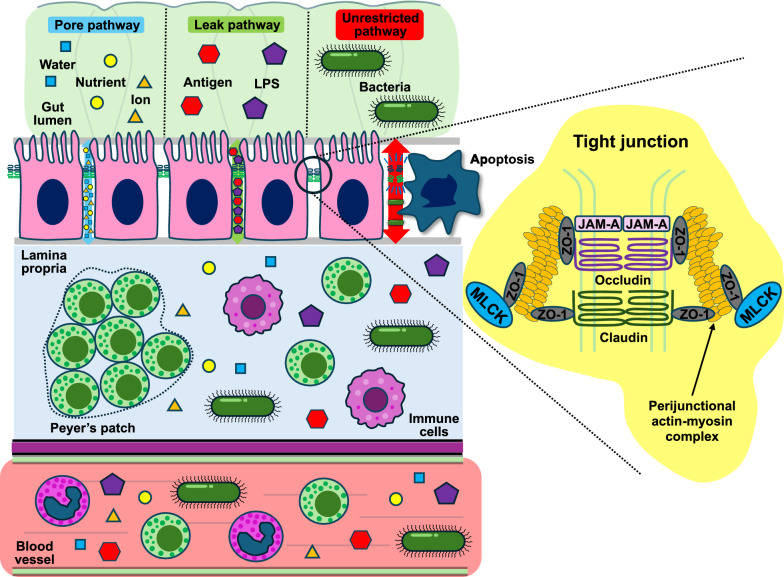
Schematic representation of gut barrier dysfunction in critical illness. Under basal conditions, TJs, composed of key proteins such as members of the claudin family, occludin, ZO-1, and JAM-A, and the perijunctional actin-myosin ring, maintain gut integrity and prevent the translocation of harmful substances. Activation of MLCK enhances the phosphorylation of perijunctional myosin light chains, ensuring cytoskeletal contraction and the subsequent opening of TJs. During critical illness, TJs are compromised, leading to increased gut permeability through three distinct pathways. The pore pathway allows passage of small ions and water, while the leak pathway allows larger molecules such as lipopolysaccharide (LPS). Additionally, the TJ-independent unrestricted pathway occurs at areas of epithelial damage enabling the translocation of whole bacteria. These changes are often accompanied by a shift in the microbial community towards dysbiosis with invasive pathogens. The disruption of barrier function increases the chance of microbes and microbial-derived products having distant effects, potentially triggering systemic inflammation through mucosal immune activation and leading to organ dysfunction

The gut comprised trillions of microorganisms, a single cell layer epithelial barrier separating the microbiome from the host, and the mucosal immune system. Under basal conditions, each of these elements of the gut interact with each other to maintain homeostasis in a manner that is beneficial to both the host and its inner microbial community. In health, the gut selectively absorbs nutrients and helps shape systemic immunity while simultaneously preventing the translocation of harmful pathogens and toxins into the systemic circulation [1]. However, during sepsis, trauma, burn injury, and other forms of critical illness, this finely tuned system is often disrupted with profound alterations in the gut microbiome with disease-promoting features, increased gut permeability through altered expression of tight junction (TJ) proteins, and dysregulated immune activation in response to overwhelming stimuli (Fig. 1) [2]. Dysbiosis, with a shift from a protective to a pathological microbiome, often referred to as a “pathobiome”, further exacerbates the risk of gut-derived sepsis [3, 4]. These changes propagate a maladaptive inflammatory response, potentially leading to the progression of multiple organ dysfunction associated with increased morbidity and mortality. Consequently, the gut has been hypothesized to be the “motor” of critical illness for decades [5–7]. Among multiple elements of gut integrity, intestinal hyperpermeability and an altered microbiome have emerged as being closely associated with—and possibly causative of—adverse clinical outcomes in the context of critical illness.

Both critical illness-induced gut hyperpermeability and dysbiosis represent potential target for therapeutic interventions. By evaluating current evidence from both clinical and pre-clinical studies, this review will examine mechanisms underlying gut barrier dysfunction, the role of the microbiome in modulating the host immune response, and the potential for targeting these elements to improve outcomes in critically ill patients [8].

## The healthy gut

### The intestinal epithelium

The intestinal epithelium functions as the primary barrier between the host and the complex microbial community within the gut lumen [9]. The intestinal epithelium comprised just a single layer of cells; however, it has a vast surface area, estimated to be 32 square meters [9]. A protective mucus layer covers the intestinal epithelium, preventing direct contact between the epithelial surface and luminal contents. Pluripotent stem cells near the base of the crypt give rise to daughter cells which differentiate into absorptive enterocytes, mucus-producing goblet cells, enteroendocrine cells, tuft cells which initiate mucosal immunity against parasitic infections and defensin-producing Paneth cells [7]. With the exception of Paneth cells which migrate to the crypt base, all other epithelial cells gradually migrate up to the tips of the villi. Epithelial cells are either extruded whole into the gut lumen or die by apoptosis ensuring continuous turnover and renewal of the intestinal epithelium. The entire journey from crypt base to villus tip takes 3–5 days [10]. As such, maintenance of gut integrity is crucial for preserving host homeostasis and defending against invasive pathogens [1].

Adjacent epithelial cells are tightly connected through TJs, desmosomes, and adherent junctions, forming a cohesive and robust barrier. This barrier is not only physical, but also functional, selectively allowing the absorption of essential nutrients while blocking the entry of harmful substances, such as pathogens and toxins (including endotoxin), into the bloodstream. Additionally, while outside the scope of this review, the gut microcirculation functions in food absorption and metabolic substance exchanges and its dysregulation has been shown to play a significant role in critical illness [11].

Transport of luminal products across the epithelium can occur through either transcellular or paracellular routes. Transcellular transit moves substances through cells via transmembrane transporters such as glucose transporter or sodium-glucose linked transporter. In contrast, paracellular transit moves substances between cells. TJs connect adjacent epithelial cells and limit the free passage of substances through gaps on the apical side. Paracellular transport is controlled by two TJ-dependent and one TJ-independent pathway [12]. The high-capacity, size and charge-selective pore pathway facilitates the selective passage of small (less than 7 Å diameter) molecules across the epithelial barrier. The low-capacity, nonselective leak pathway allows the passage of larger molecules (up to 100 Å diameter), such as lipopolysaccharides. The TJ-independent unrestricted pathway occurs at areas of intestinal damage and does not have a size limit. While bacteria are too large to translocate through either the pore or leak pathway, they can pass from the gut lumen through the unrestricted pathway to ultimately gain access to the host’s lamina propria or intravascular space.

Different TJ proteins regulate the pore and leak pathways. The claudin family is primarily responsible for regulating the pore pathway. Pore-forming proteins, such as claudin-2 and claudin-15 mediate the paracellular transport of small cations, such as sodium and water, while other members of the claudin family function as sealing proteins [13, 14]. The leak pathway is mediated by a number of different TJ proteins including occludin, zonula occludens (ZO)-1 and junctional adhesion molecule (JAM)-A. The apical TJ is functionally and structurally linked to the perijunctional actin-myosin ring. Myosin light chain kinase (MLCK) phosphorylates the perijunctional myosin regulatory light chain, resulting in contraction of the actin-myosin ring and opening of TJs with increased leak permeability [12].

### Gut microbiome

The gut hosts a vast and diverse community of microorganisms, and metagenomic analyses using 16S rRNA sequencing have revealed the full scope of the gut microbiome [15]. The gut microbiome comprise bacteria, viruses, fungi, and other microbes, collectively estimated to number approximately 40 trillion microorganisms—roughly equivalent to the total number of human cells in the body [16, 17]. These microorganisms are not merely passive residents. Instead, they play an active and crucial role in maintaining host health and functionality by directly and indirectly impacting nearly every organ system. Under basal conditions, the gut microbiota and host maintain a mutually beneficial relationship. The microbiota contribute to numerous physiological processes, including fermentation of indigestible carbohydrates, synthesis of essential vitamins, and regulation of metabolic and immune functions. In return, the host provides a nutrient-rich environment and protection for the microbiome. Microbial products, such as essential short-chain fatty acids (SCFAs), serve as the primary energy source for intestinal epithelial cells and promote the growth of beneficial gut microbes [18]. This symbiotic relationship is fundamental for maintaining gut homeostasis and, by extension, overall health.

However, the stability of the gut microbiome can be disrupted by various factors, including diet, stress, medications, and illness. When this disruption occurs, the composition of the microbiome may shift towards a dysbiotic state [19–21]. Dysbiosis, characterized by reduced diversity and the overgrowth of pathogenic species, has been implicated in a wide range of chronic illness, including obesity, diabetes, and inflammatory bowel disease [22]. Importantly the gut microbiome communicates with multiple distant organs through several axes including the kidneys, heart, brain, lung, and liver (reviewed in [23]).

### Intestinal immune system

In the intestinal lumen, mucus secreted by goblet cells and antimicrobial peptides produced by Paneth cells, act as physical and biological barriers to the overgrowth of pathogenic bacteria [24, 25]. In addition, an extensive network of immune cells lies beneath the epithelial surface. The gut-associated lymphoid tissue, which includes intraepithelial lymphocytes, lamina propria immune cells, Peyer's patches, and mesenteric lymph nodes, is responsible for orchestrating immune responses to the diverse antigens encountered in the gut, and the gut has more lymphocytes than any other location in the body. In addition, secretory immunoglobulin A (IgA), which originates from plasma cells in germinal centers, binds to and coats commensal bacteria [26]. This intricate immune system is finely tuned to tolerate commensal microbes and dietary antigens while also defending against potential pathogens by activating both the innate and adaptive immunity [27, 28]. Therefore, the gut immune system is crucial for maintaining the balance between immune tolerance and activation. Furthermore, the gut microbiome plays an essential role in the development of immune system, as demonstrated by germ-free mice, which lack a mature host defense and are more susceptible to pathogens [29, 30].

## Gut permeability in critical illness

Critical illness profoundly disrupts gut integrity by increasing permeability, accelerating epithelial apoptosis, reducing cell proliferation, and slowing cell migration. When critical illness causes the gut to become “leaky”, this can, in turn potentially worsen disease severity as microbial products and less commonly intact microbes can translocate via TJ-dependent and independent-pathways into the systemic circulation, propagating inflammation and immune activation. This inflammatory response contributes to endothelial dysfunction, capillary leakage, and further perturbation of an increasingly dysregulated immune system [5–7, 10].

Alterations in TJ proteins have been identified in pre-clinical models within one hour of the onset of sepsis [31]. This contributes to worsened barrier function as animal models of abdominal sepsis and *Pseudomonas aeruginosa* pneumonia both demonstrate hyperpermeability through the pore, leak and unrestricted pathways [31]. Notably increased permeability occurs as early as 6 h after the onset of sepsis [31]. Gut hyperpermeability has also been identified in critically ill patients as absorption rates of lactulose, a compound minimally absorbed in healthy intestines has been demonstrated to be elevated in intensive care unit (ICU) patients [32]. Further, a multisugar test for identifying intestinal permeability in mechanically ventilated patients on enteral nutrition for at least 3 days demonstrates that permeability can be measured and is altered at the whole gut level and in the small bowel [33]. Notably, impaired renal function reduced the reliability of this measurement in critically ill patients.

The mechanisms through which critical illness induce gut hyperpermeability are complex. In response to pathophysiological stimuli, inflammatory mediators can modulate barrier function by altering the expression and localization of TJs. This shift causes the uncontrolled movement of numerous molecules, further destabilizing the gut environment. As an example of interactions between cytokines and TJ proteins, interleukin (IL)-13 and IL-22 enhance the transcription and expression of claudin-2 in the intestinal epithelium, increasing permeability through the pore pathway. Additionally, inflammatory cytokines such as IL-1β and tumor necrosis factor (TNF) activate the leak pathway through upregulating MLCK expression [12, 34]. Such cytokine-induced alteration in TJ expression promotes immune activation and tissue inflammation, playing a critical role in the onset and progression of various intestinal and systemic diseases [35]. Acceleration of intestinal apoptosis through death-receptor and mitochondrial pathways in response to mucosal damage further exacerbates barrier dysfunction via the unrestricted pathway and local inflammation [36].

Pre-clinical studies of sepsis demonstrate increased permeability is associated with upregulation of jejunal claudin-2 and claudin-5, along with downregulation of occludin and JAM-A [31]. Further subcellular localization of claudins 1, 3, 4, 5, and 8 changed from being localized on the microvillous surface of epithelial cells and along the lateral membranes of these cells to a diffuse distribution in a model of intra-abdominal sepsis, and colonic claudin-2 expression is simultaneously markedly upregulated, correlating with increased intestinal permeability [37]. Further, ileal claudin-2 expression increased in samples from patients with sepsis from abdominal perforation compared to age and sex matched patients who underwent elective intestinal resection while claudin-15, another pore-forming protein decreased [38].

Pre-existing comorbidities substantially impact clinical outcomes in critical illness. For example, in patients with sepsis and community-acquired pneumonia, alcohol use disorder is associated with longer length of hospital stay and higher mortality [39, 40]. Notably, chronic alcohol use also induces disrupted gut barrier function and dysregulated inflammation in sepsis [41–43]. Notably, chronic ethanol ingestion increases permeability across all three pathways—pore, leak, and unrestricted—and this is associated with increased mortality in a pre-clinical model of intra-abdominal sepsis [44]. In cancer patients, particularly those with hematologic malignancies, treatments such as antibiotics and chemoradiation increase the risk of intestinal hyperpermeability [45]. Additionally, cancer animal models exhibit increased intestinal apoptosis and disrupted gut integrity following sepsis compared to previously healthy animals subjected to the same insult, demonstrating the deleterious effect of cancer alone in the absence of treatment [46, 47].

It is important to note that while permeability is increased and TJs are altered in both human studies and pre-clinical animal studies of sepsis, these associations cannot prove that hyperpermeability and TJ dysregulation are harmful in critical illness. In order to test the functional significance of these findings, mechanistic pre-clinical studies have therefore examined the role of altered TJs in sepsis. First, the role of upregulated claudin-2 seen in septic patients was examined in mice with intra-abdominal sepsis [38]. Similar to findings in patients, claudin-2 expression was upregulated in the gut of septic mice. Systemic deletion of claudin-2 resulted in decreased gut permeability and reduced intestinal inflammation with lower T cell activation in the intraepithelial compartment and Peyer's patches. Notably, claudin-2 deletion improved survival in murine sepsis demonstrating a mechanistic link between TJ alteration and mortality.

Further, in light of pre-clinical studies demonstrating alterations in occludin levels following sepsis, mice with occludin knocked out specifically in their intestinal epithelium were compared to wild-type mice after intra-abdominal sepsis [48]. Animals with intestine-specific occludin deletion had a significant increase in gut leak permeability associated with increased levels of the TJ ZO-1. Further, knockout mice had significant increases in IL-6 and MCP-1 systemically as well as increased bacterial burden. Knockout mice also had higher levels of IL-1β and MCP-1 in the jejunum in addition to increased MCP-1 and IL-17A in the peritoneal fluid. These differences were associated with worsened survival in mice lacking occludin in their intestinal epithelium, also demonstrating a mechanistic link between gut TJs and mortality in septic hosts.

Given its role in mediating the leak pathway of permeability, the role of MLCK in modulating gut barrier function and survival in sepsis has also been examined with conflicting results. MLCK-deficient mice have improved intestinal permeability with increased levels of ZO-1 and claudin-15, associated with significantly improved survival rates following intra-abdominal sepsis [49, 50]. Further, pharmacological MLCK inhibition mitigates intestinal permeability, mucosal damage, and TJ protein alterations following severe burn injury [51]. In contrast, MLCK worsens lung permeability and increases mortality following *Pseudomonas aeruginosa* pneumonia-induced sepsis [52]. Further, pharmacologic inhibition of MLCK paradoxically worsens intestinal leak pathway permeability with increased phosphorylated MLC following polymicrobial sepsis, associated with increased mortality [53].

Due to diverse expression of TJ proteins, altering them may result in phenotypes unrelated to their role in gut permeability. The TJ protein JAM-A is increased in sepsis. Genetic deletion if JAM-A leads to increase gut permeability as might be expected [54]. However, JAM-A deletion is paradoxically associated with decreased bacteremia, systemic TNF and IL-1β expression and notably improved survival. This survival change appears to be independent of gut permeability as this is not present in mice lacking JAM-A in the intestine. Further experiments demonstrate a role of JAM-A on both neutrophils and opsonizing IgA in mediating the mortality benefit in mice lacking JAM-A, showing that studies must carefully examine multiple tissue types and processes prior to ascribing changes in mortality specifically to changes in gut epithelial permeability, even when sepsis-specific difference in barrier function are present.

While direct evidence of bacterial translocation is challenging to demonstrate clinically under most circumstances, the crucial role of increased gut permeability in aggravating organ dysfunction remains widely recognized as leakage of luminal contents can occur without intact bacteria translocating [55, 56]. Unlike the pore and leak pathway, the unrestricted pathway of permeability is not TJ-dependent and occurs at areas of epithelial damage such as by increased sepsis-induced gut epithelial apoptosis [12, 34]. There is no size restriction to molecules moving through the unrestricted pathway. Highlighting the potential of targeting gut epithelial apoptosis, gut-specific overexpression of the anti-apoptotic Bcl-2 in transgenic mice not only reduced sepsis-induced cell death, but it also reduced hyperpermeability and improves survival following intra-abdominal sepsis [57, 58].

## The microbiome in critical illness

During critical illness, the composition of the gut microbiome undergoes profound changes that can have significant clinical consequences. Beyond pathological factors induced by critical illness, therapeutic interventions intended to improve outcome in the ICU—including (but not limited to) antibiotics, opioids, anti-delirium medications and proton pump inhibitors—also promote dysbiosis [59]. One of the most notable changes observed in critically ill patients is a marked loss of microbial diversity with changes occurring as early as 6 h of the onset of critical illness [60–62]. In healthy individuals, the gut microbiome is a complex, diverse ecosystem with no single dominant genus. However, critical illness frequently leads to a reduction in this diversity, resulting in low or ultra-low diversity communities. Research has shown that, in some cases, a single bacterial genus can dominate more than 50% of the gut microbiome in critically ill patients [63]. This decline in microbial diversity is also found outside the intestine. In critically ill patients on mechanical ventilation, the upper and lower respiratory tract become dominated by gut-associated bacteria, with decreasing microbial diversity within 24 h of intubation [64]. The interaction with the gut extends beyond the lungs; gut-associated bacteria have also been found to be enriched in the brains of mice 5 days after the onset of abdominal sepsis [65].

In critically ill patients, the gut microbiome can shift from a symbiotic community that supports health to a “pathobiome” that contributes to diseases. This pathobiome is characterized by an overgrowth of opportunistic pathogens, such as *Enterococcus*, *Staphylococcus*, and *Pseudomonas*, which are normally present in low numbers but can become dominant under critical illness, leading to nosocomial infection [66, 67] or exacerbating systemic inflammation. Furthermore, the disrupted composition of microbiome is associated with higher short-term and long-term mortality in critically ill patients [68, 69]. Beyond changes in microbial composition, critical illness also disrupts the balance of metabolic functions in the gut. The loss of a protective microbiome impairs the production of SCFAs, which are crucial for maintaining gut barrier integrity, while increasing the production of harmful metabolites that can exacerbate inflammation [70–73].

Multiple clinical studies have shown associations between microbiome changes and patient outcomes, but correlation is clearly not the same as causation. To address this challenge, pre-clinical models have provided valuable insights into the causal relationship between the microbiome and consequences in critical illness. Genetically identical mice from different vendors, with distinct microbiomes, display different survival rates when subjected to sepsis with mice with a more diverse and balanced microbiome exhibiting better survival rates. These differences in both the microbiome and survival are associated with numerous differences in the host immune responses. After co-housing mouse from different vendors for 3 weeks, their microbiomes become similar. This allows for an examination of whether the microbiome is mechanistically responsible for differences in mortality in genetically identical mice as well as changes in the immune response. Notably, once mice from different vendors are co-housed and develop a common microbiome similar to mice that have a more complex microbiome at baseline, survival is similar and host immune response is similar, regardless of initial vendor (and microbiome) [74]. These experiments provide powerful evidence that the gut microbiome plays a causal role in determining the outcome of pre-clinical critical illness and that modulating the microbiome could be a potential therapeutic strategy in critically ill patients.

## Interactions between intestinal epithelia, microbiome, and immune system

### The impact of gut barrier dysfunction on microbiome and immune system

Disrupted intestinal barrier function significantly impacts the immune system and microbiome through alterations of TJ proteins in chronic disease. Gut barrier dysfunction, driven by increased MLCK activity, has been shown to be insufficient to induce experimental colitis on its own; however, a broadly activated mucosal immune response intensified the onset and severity of immune-mediated colitis [75]. In addition, transgenic mice that express constitutively active MLCK activity have increased intraepithelial bacteria, dysbiosis and more severe colitis compared with wild-type mice. Intriguingly, co-housing wild-type mice with transgenic mice leads to dysbiosis and emergence of colitis in wild type, suggesting that intestinal barrier dysfunction is a possible cause of dysbiosis and immune activation [76].

Pre-clinical sepsis models have also highlighted the complex relationship between gut barrier dysfunction, immune regulation, and the microbiome in critical illness. Decreased gut permeability in claudin-2 knockout mice is associated with both diminished immune activation and a less pathogenic microbiome. Although relative abundances of *Bacteroidetes* and *Firmicutes* are similar between wild type and claudin-2 knockout mice at baseline, the latter have a more pronounced increase in *Firmicutes* following intra-abdominal sepsis. The functional significance of this altered microbiome is demonstrated by experiments showing improved survival rates in mice injected with cecal slurry from septic claudin-2 knockout mice compared to cecal slurry from septic wild-type mice. These findings suggest that claudin-2 expression may play a pathophysiological role in shaping gut microbiome composition, potentially through modulation of immune response or altered molecular transport [38].

### Microbiome-driven immunomodulation

The gut microbiome and immune system maintain a mutually beneficial relationship, where bacteria stimulate host immune response and contribute to immune system development. For example, bacteriocins from gut bacteria prompt Paneth cells to produce antimicrobial peptides, preventing the overgrowth of harmful bacteria [77]. Commensal bacteria also influence immune cell development, as seen in germ-free mice which have smaller mesenteric lymph nodes, Peyer's patches, and fewer numbers of IL-17-producing T helper 17 (Th17) cells and regulatory T (Treg) cells than mice with an intact microbiota. Th17 cells recruit neutrophils to combat infections, while Tregs suppress excessive inflammation, preventing conditions such as inflammatory bowel disease [78]. Specific bacteria promote the development of mucosal immune cells. While segmented filamentous bacteria are essential for the appearance of Th17 cells in lamina propria, *Bacteroides fragilis* and *Clostridioides* are involved in the development of Tregs, counteracting Th17 responses [79, 80]. Additionally, IgA antibodies, produced in response to commensal and pathogenic bacteria, help maintain a stable microbial environment, preventing pathogenic colonization and inflammation [81–83].

Experimental evidence also demonstrates that alterations in the gut microbiome directly modulate the host immune response in pre-clinical models of critical illness. Septic mice with different microbiomes exhibit unique profiles of immune cell activation, particularly in CD4 + and CD8 + T cells. Notably, co-housing these mice eliminates all immunophenotypic differences, indicating that immune variations are associated with the composition of gut microbiome [74]. Further introduction of specific microbial communities into germ-free or antibiotic-treated mice modulate the immune response to sepsis. Mice colonized with a microbiome rich in SCFA-producing bacteria have enhanced gut barrier function and a more regulated immune response, leading to improved survival in sepsis [84].

Importantly, the relationship between disrupted gut microbiota and dysregulated immune response has also been examined in a longitudinal cohort of critically ill patients in an integrated system-level analysis using 16S rRNA sequencing of stool samples and single-cell RNA profiling of blood. This study revealed that the gut microbiome and systemic immunity function as an integrated metasystem. Dysbiosis, characterized by *Enterobacteriaceae* enrichment, is associated with impaired innate immune responses, such as dysfunctional neutrophils, and increased susceptibility to infections [67]. These findings suggest that disruptions in the gut-immune metasystem may drive impaired host defense and higher infection risk in critical illness.

## Modulation of gut integrity

Given the critical role of gut permeability in the pathogenesis of critical illness, modulation of gut permeability is a key potential therapeutic target. Several interventions aimed at preserving or restoring gut integrity have been proposed that may offer clinical benefits (Table 1).

**Table 1:** Therapeutic interventions focusing on gut permeability and microbiome in critical illness

Therapeutic approach	Interventions	Description	Advantages	Disadvantages	Grade of evidence
Modulation of gut permeability	EGF	EGF promotes healing of intestinal epithelial cells and reduces permeability	Mucosal healing and reduced inflammation	No clinical data in critical illness	Pre-clinical models [94, 95]
	MLCK inhibitor	MLCK inhibitors reduce intestinal permeability by stabilizing tight junctions between cells	Decreased gut permeability and mitigated systemic inflammation	Early-stage basic research, possible detrimental effect in sepsis	Pre-clinical models [51, 53, 85]
	Caspase inhibitor	Inhibition of apoptosis in intestinal cells, reducing epithelial damage and permeability	Protection against gut barrier dysfunction in sepsis	No clinical data	Pre-clinical models [90–92]
Microbiome-targeted therapies	Prebiotics	Non-digestible fibers promoting the growth of beneficial gut microbiota	Enhanced growth of beneficial bacteria and potentially improved gut barrier function	Evidence of efficacy in critical illness is limited, and overgrowth of certain bacteria is possible	Limited clinical trials [104, 106, 107]
	Probiotics	Live microorganisms that provide health benefits by restoring normal gut flora	Lower rate of infectious complications, improved gut barrier function, and reduced inflammation	Variability in strain efficacy and risk of infection in immunocompromised patients	Mixed evidence from clinical trials [99, 100]
	Synbiotics	Combination of probiotics and prebiotics	Potential improvement of gut microbiota balance and immune function	Complex to administer, potential for adverse interactions between components	Early clinical trials [108, 109]
	FMT	Transplantation of stool from a healthy donor to restore normal gut flora in a patient	Efficacy in treating recurrent *Clostridioides difficile* infections and potential for restoring gut balance	Limited data in critical illness, risk of transferring infections or resistant bacteria	Pre-clinical models, case reports [112, 113]
	Enteral nutrition	Enteral feeding strategies that aim to support gut health and microbiota balance	Enhancement of gut integrity, reduced inflammation, and improved clinical outcomes	Impact of specific nutrition on gut microbiome remains to be explored	Clinical trials, standard of ICU care [110]
	SDD	Use of non-absorbable antibiotics to reduce pathogenic bacteria in the gut	Reduced rate of infectious complications in ICU patients	Risk of antibiotic resistance, impact on overall microbiome is unclear	Clinical trials, meta-analyses [116, 117, 130]

*EGF* epidermal growth factor, *MLCK* myosin light chain kinase, *SDD* selective decontamination of the digestive tract, *FMT* fecal microbiota transplant, *ICU* intensive care unit

### Epithelial-based strategies

1. TJ modulation
  Directly targeting TJs in the gut is challenging in light of the fact that many TJ proteins are expressed in multiple different tissues, and therapeutic approaches run the risk of off target effects. As an example, genetic deletion of MLCK, which regulates the contraction of the actin-myosin ring controlling gut permeability, reduces intestinal leakiness and systemic inflammation in animal studies [49]. To translate the effect of MLCK inhibition into clinical practice, the impact of membrane permeant inhibitor of MLCK (PIK) was investigated using pre-clinical models. PIK alleviates the reduced expression of occludin and ZO-1 following ethanol exposure and burn injury [85]. However, in a murine model of sepsis, PIK unexpectedly leads to worse survival and increased intestinal hyperpermeability, suggesting caution when targeting the gut barrier as a therapeutic approach in sepsis [53]. Further, ML-7, which targets the ATP binding site of MLCK, fails to impact survival in a murine model of sepsis [77]. These differing results between genetic deletion and pharmacologic inhibition studies might be explained by the fact that MLCK is also expressed in the smooth muscle, vascular endothelia, and epithelia in the other tissues [78]. To address the numerous additional undesired effects of PIK and ML-7, the small molecule inhibitor divertin was developed, which prevents MLCK1 recruitment without inhibiting its enzymatic activity. Divertin effectively blocks barrier loss and disease progression in inflammatory bowel disease [86], and, as such, potentially represents a promising new approach to safely target specific enzyme functions in critical illness as well.
  While there are currently no available drugs directly modulating claudin-2, casein kinase 2 (CK2) inhibition can inactivate claudin-2 channels in vitro and in vivo among its numerous effects [87]. The CK-2 inhibitor silmitasertib, significantly reduced mortality in mice following cecal ligation and puncture [88]; however, it did not alter either dysbiosis or survival in a different mouse sepsis study using a similar model [38].
2. Inhibition of epithelial apoptosis
  The unrestricted pathway of gut permeability, which allows the passage of large molecules and bacteria due to epithelial cell damage or apoptosis, presents another therapeutic target. Strategies to reduce apoptosis, such as the overexpression of anti-apoptotic proteins such as Bcl-2 in the gut epithelium, have demonstrated protective effects in pre-clinical sepsis models [57, 58, 89]. There are no inhibitor strategies that effect only the gut epithelium. Strategies to decrease apoptosis would also impact other cell types where sepsis alters apoptosis such as lymphocytes, dendritic cells and neutrophils. The caspase inhibitor z-VAD, prevents apoptosis and barrier dysfunction in intestinal cell lines [90] and prevents epithelial apoptosis, gut hyperpermeability, and local inflammation in murine models of intestinal ischemia–reperfusion or severe acute pancreatitis [91, 92]. This inhibitor improves survival in a model of intra-abdominal sepsis associated with decreased lymphocyte apoptosis [93], although whether this survival advantage is related to gut epithelial apoptosis is not known.
3. Regulator of epithelial cells
  Epidermal growth factor (EGF) impacts gut integrity in numerous different ways. Systemic EGF treatment decreases apoptosis, improves proliferation, restores villus length and improves permeability in both intra-abdominal sepsis and pneumonia, even if initiated 24 h after septic insult. Notably, systemic treatment with EGF improves survival following pre-clinical sepsis [94, 95]. Further, intestine-specific overexpression of EGF in transgenic mice also improves gut integrity and improves survival [96], demonstrating that targeting gut epithelial integrity is a novel pre-clinical therapeutic approach towards improving outcomes in sepsis. Another class of potential therapeutic agents that target gut integrity is glucagon-like peptide-2 analogs. These promote intestinal growth and mucosal repair and have shown promise in reducing gut permeability in animal models [97]. Additionally, agents targeting inflammatory pathways involved in gut barrier disruption, such as TNF inhibitors, are being explored for their potential to preserve gut integrity during critical illness, with the understanding that they also have numerous effects independent of modulation of gut integrity [98].

### Microbiome-based strategies

Considering the crucial role of the microbiome in modulating immune responses and potentially influencing outcomes in critical illness, therapeutic interventions to alter the microbiome could also be a promising strategy. Notably, the strategies outlined below have all been used in critically ill patients (some more widely than others) and thus are much closer to changing clinical practice at the bedside than targeting epithelial integrity (Table 1):

1. Probiotics, prebiotics, and synbiotics
  Probiotics introduce live microbes to restore microbiota balance in compromised states with a goal of preventing subsequent infections. The largest randomized controlled trial of probiotics failed to demonstrate a significant benefit in reducing the rate of ventilator associated pneumonia in critically ill patients [99]. Multiple meta-analyses have been published on the role of probiotics in patients in the ICU [100–102]. These differ somewhat in conclusions based upon inclusion or exclusion of higher risk of bias studies. There is strong agreement that probiotics do not improve hospital mortality, diarrhea, hospital acquired pneumonia or *C. difficile* colitis. While inclusion of all studies demonstrates a benefit of probiotics on ventilator-associated pneumonia and hospital length of stay, sensitivity analyses without high risk of bias negates these beneficial effects. Additionally, bacteremia following probiotic administration has been shown to be a concerning side effect in a small number of patients [103].
  Prebiotics are vital supplements that promote the growth of beneficial microbiota by supplying nutrients, such as carbohydrates and fiber, which help maintain intestinal barrier integrity. Pre-clinical sepsis studies in mice have shown that a fiber-rich diet enhances survival and promotes beneficial genera such as *Akkermansia* and *Lachnospiraceae*, although these effects can be negated by antibiotic use [104]. Fiber plays a critical role in maintaining gut health by serving as a substrate for beneficial bacteria; a high-fiber diet helps preserve the intestinal mucus layer [105], whereas low-fiber intake can lead to microbial instability and the proliferation of harmful strains [22]. However, significant benefits of fiber in critically ill patients on patient-centric outcomes have not been demonstrated [106, 107].
  Synbiotics, a combination of prebiotics and probiotics, have been found to synergistically improve gut microbiota [108, 109]. Studies on synbiotics in patients are relatively limited, and their results have been inconsistent, possibly due to variations in strains, timing of administration, and treatment duration. Consequently, current nutritional guidelines do not recommend the routine use of probiotics, prebiotics, or synbiotics in ICU settings [110]. Further research is needed to determine what (if any) role these interventions play in improving outcomes in critically ill patients including evaluating the most effective strains and dosing regimens as well as patients most likely to respond to treatment.
2. Fecal microbiota transplantation (FMT)
  FMT is a procedure in which stool from a healthy donor is transferred to a patient to restore a balanced and diverse microbiome. FMT has remarkable efficacy in clinical trials outside the ICU in patients with recurrent *C. difficile* infection and has been studied in numerous other clinical settings [111]. Pre-clinical sepsis models demonstrate that FMT improves survival by enhancing pathogen clearance via restoring host immunity in an interferon regulatory factor 3-dependent manner, linked to expansion of butyrate-producing *Bacteroidetes* [112]. In contrast, data in critically ill patients are limited to a small number of case studies due to a number of potential barriers to FMT including commitment to withholding antibiotics (as these would be expected to alter the microbiome following transplant) as well as unknown impact of giving bacteria to a patient with an already altered microbiome who is likely immunosuppressed [113, 114].
3. Selective decontamination of the digestive tract (SDD)
  SDD aims to eliminate pathogenic bacteria from the intestinal tract in mechanically ventilated patients using a combination of oral and systemic antibiotics [115]. SDD has been extensively studied in critical illness, and meta-analyses suggest SDD reduces mortality and respiratory infections in critically ill patients on mechanical ventilation [116]. The preponderance of this evidence comes from patients in settings with low antibiotic resistance. Conversely, trials in settings with moderate-to-high resistance have not shown such benefits, raising concerns about its efficacy in these patient populations [117]. These disparate results have led to significant variance in the use of SDD with the majority of ICUs around the world never using it but a subset of countries in which it is considered standard of care.
4. Enteral nutrition
  The gut microbiome is profoundly influenced by various types of nutrients under homeostatic conditions. Diets rich in proteins and animal fats tend to promote *Bacteroides*, while carbohydrate-heavy diets favor *Prevotella* [118]. High-fat diets, in particular, can disrupt microbial balance, leading to a decrease in *Bacteroidetes* and an increase in *Firmicutes* and *Proteobacteria* [119]. Furthermore, studies indicate that excessive fat intake is associated with increased proportions of *Firmicutes* and *Proteobacteria*, which have been linked to obesity in animal models, while weight loss through dietary restriction can help restore the microbiome to its original state [119].
  Parenteral nutrition, commonly used in critical illness, alters the microbiome by promoting dysbiosis and increasing *Proteobacteria*, although partial enteral nutrition can mitigate some of these adverse effects [120, 121]. While the connection between nutrition and the microbiome is well-documented in healthy individuals, the impact of specific nutritional strategies on gut microbiome in critically ill patients is a complex topic that continues to be the topic of intense research [59, 122, 123].
5. Others
  The use of broad-spectrum antibiotics in critically ill patients is often essential but can result in significant disruption to the gut microbiome, leading to dysbiosis and the overgrowth of pathogenic bacteria. Emerging strategies for preventing antibiotic-induced dysbiosis hold promise in the ICU setting. One such approach involves ribaxamase, a beta-lactamase derived from *Bacillus licheniformis*, which preserves microbiome integrity by hydrolyzing residual beta-lactams in the intestinal tract without affecting drug pharmacokinetics [124]. Phase II studies have shown that ribaxamase may reduce the risk of *C. difficile* infection when administered alongside intravenous ceftriaxone [125]. Further, lolamicin is a Gram-negative specific antibiotic that targets the lipoprotein transport system [126]. Lolamicin has activity against more than 130 multidrug-resistant isolates and has efficacy in numerous models of sepsis while simultaneously sparing the gut microbiome due to low sequence homology in pathogenic bacteria versus commensals. Another innovative therapy, DaV-132, uses activated charcoal to absorb residual antibiotics in the colon [127, 128]. Additionally, antibiotic stewardship programs, which focus on optimizing antibiotic use by selecting the most appropriate agent, dose, and duration, are crucial for minimizing the negative impact of antibiotics on the microbiome [129].

## Conclusions

**Fig. 2:**
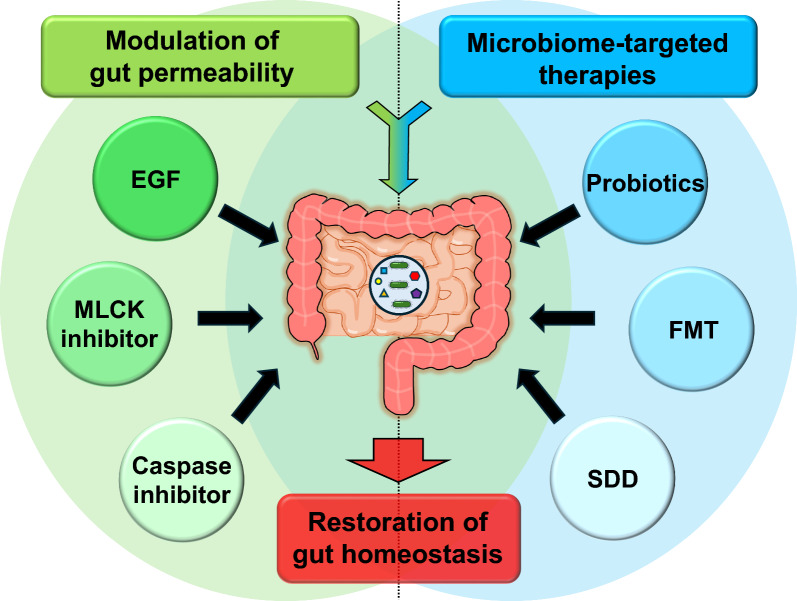
Potential therapeutic interventions modulating gut permeability and targeting the microbiome in critical illness. Pharmacological approaches such as EGF, MLCK inhibitors, and caspase inhibitors, can potentially modulate gut permeability by strengthening epithelial TJs and preventing apoptosis (left side of figure). Microbiome-targeted therapies, including probiotics, FMT, and SDD can also potentially restore a healthy microbiome (right side of figure)

The therapeutic implications of gut permeability and microbiome modulation in critical illness represent rapidly evolving fields (Fig. 2). Currently, there are no readily translatable therapies available at the bedside to target gut permeability. In contrast, there are multiple treatments that target the gut microbiome. Of these, SDD has been the most extensively studied and is standard of care in certain locations with low risk of antimicrobial resistance although this strategy is much more controversial in settings with higher risk of antimicrobial resistance. Additionally, while some clinicians use probiotics in the ICU, data on their potential efficacy are both conflicting and of low certainty. Future research should focus on translating promising pre-clinical findings into clinical practice, identifying the most effective interventions, and determining the optimal timing and patient populations for these therapies. Additionally, personalized approaches that take into account the individual patient's microbiome and gut barrier status may offer the greatest potential for improving outcomes in critically ill patients.

As our understanding of the gut integrity in critical illness continues to grow, novel therapeutic strategies are likely to emerge, offering new hope for patients facing life-threatening conditions. The integration of gut-targeted therapies into standard critical care practice potentially holds the promise of reducing morbidity and mortality, ultimately improving the quality of care for critically ill patients.

## Data Availability

Not applicable.

## Abbreviations

TJ: Tight junction
ZO: Zonula occludens
JAM: Junctional adhesion molecule
MLCK: Myosin light chain kinase
IL: Interleukin
TNF: Tumor necrosis factor
SCFA: Short-chain fatty acid
IgA: Immunoglobulin A
CLP: Cecal ligation and puncture
ICU: Intensive care unit
KO: Knockout
EGF: Epidermal growth factor
PIK: Membrane permeant inhibitor of myosin light chain kinase
FMT: Fecal microbiota transplantation
SDD: Selective digestive decontamination
